# Getting to the Core: Exploring the Embryonic Development from Notochord to Nucleus Pulposus

**DOI:** 10.3390/jdb12030018

**Published:** 2024-07-03

**Authors:** Luca Ambrosio, Jordy Schol, Clara Ruiz-Fernández, Shota Tamagawa, Kieran Joyce, Akira Nomura, Elisabetta de Rinaldis, Daisuke Sakai, Rocco Papalia, Gianluca Vadalà, Vincenzo Denaro

**Affiliations:** 1 Operative Research Unit of Orthopaedic and Trauma Surgery, Fondazione Policlinico Universitario Campus Bio-Medico, 00128 Rome, Italy; l.ambrosio@unicampus.it (L.A.); r.papalia@policlinicocampus.it (R.P.); denaro@policlinicocampus.it (V.D.); 2 Research Unit of Orthopaedic and Trauma Surgery, Department of Medicine and Surgery, Università Campus Bio-Medico di Roma, 01128 Rome, Italy; derinaldiselisabetta@gmail.com; 3 Department of Orthopaedic Surgery, Tokai University School of Medicine, Isehara 259-1143, Japan; schol.jordy@gmail.com (J.S.); clararuiz@tokai.ac.jp (C.R.-F.); na7896@tokai.ac.jp (A.N.); daisakai@tokai.ac.jp (D.S.); 4 Department of Medicine for Orthopaedics and Motor Organ, Juntendo University Graduate School of Medicine, Tokyo 113-8421, Japan; s-tamagawa@juntendo.ac.jp; 5 CÚRAM, SFI Research Centre for Medical Devices, University of Galway, H91 W2TY Galway, Ireland; kieran.joyce@universityofgalway.ie; 6 School of Medicine, University of Galway, H91 W2TY Galway, Ireland

**Keywords:** intervertebral disc, nucleus pulposus, embryology, degeneration, notochordal cells, development, transcription factors, regenerative medicine

## Abstract

The intervertebral disc (IVD) is the largest avascular organ of the human body and plays a fundamental role in providing the spine with its unique structural and biomechanical functions. The inner part of the IVD contains the nucleus pulposus (NP), a gel-like tissue characterized by a high content of type II collagen and proteoglycans, which is crucial for the disc’s load-bearing and shock-absorbing properties. With aging and IVD degeneration (IDD), the NP gradually loses its physiological characteristics, leading to low back pain and additional sequelae. In contrast to surrounding spinal tissues, the NP presents a distinctive embryonic development since it directly derives from the notochord. This review aims to explore the embryology of the NP, emphasizing the pivotal roles of key transcription factors, which guide the differentiation and maintenance of the NP cellular components from the notochord and surrounding sclerotome. Through an understanding of NP development, we sought to investigate the implications of the critical developmental aspects in IVD-related pathologies, such as IDD and the rare malignant chordomas. Moreover, this review discusses the therapeutic strategies targeting these pathways, including the novel regenerative approaches leveraging insights from NP development and embryology to potentially guide future treatments.

## 1. Introduction

**Figure 1:**
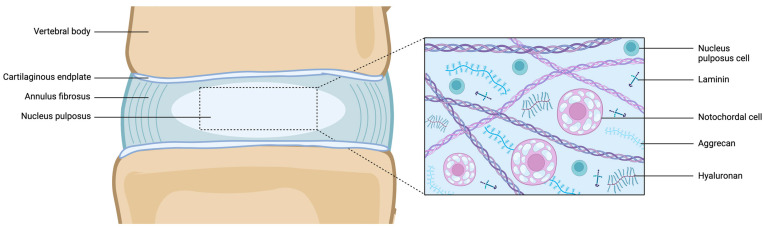
Schematic representation of the juvenile healthy IVD (left) and microscopic structure of the NP. Abbreviations: IVD = intervertebral disc; NP = nucleus pulposus. Created with BioRender.com.

The nucleus pulposus (NP) is an avascular gel-like tissue enveloped by the collagen-rich annulus fibrosus (AF) and bordered by thin superior and inferior hyaline cartilaginous endplates (CEPs), collectively comprising the three main tissues composing the intervertebral disc (IVD), a fibrocartilaginous structure situated between the vertebral bodies [1]. The extracellular matrix (ECM) plays a vital role in the biological and biomechanical functions of the NP. It primarily consists of proteoglycans (mainly aggrecan), type II collagen, and sulphated glycosaminoglycans (GAGs), such as chondroitin sulfate (Figure 1) [2].

Due to its high-water content constituting approximately 80% of its wet weight, and its composition rich in collagen, the NP, located at the innermost part of the IVD, provides load-bearing and shock-absorbing properties [3]. Unlike articular cartilage, the human NP matrix can be distinguished by its unique GAG/hydroxyproline ratio, which remains significantly higher across all ages compared to juvenile cartilage [4]. Furthermore, NP cells (NPCs) express matrix-binding proteins, particularly integrins, and exhibit a high level of integrin-laminin binding, suggesting the crucial role of laminin in cell–matrix interactions [5]. It has been suggested that distinctive markers including an aggrecan/type II collagen expression ratio >20, and the stabilized expression of hypoxia-inducible factor 1-α (HIF-1α), glucose transporter 1 (GLUT-1), sonic hedgehog (SHH), Brachyury (TBXT), keratin (KRT)-18/19, carbonic anhydrase (CA)-XII, and cluster of differentiation (CD)-24 define the phenotype of young healthy NPCs [4].

**Figure 2:**
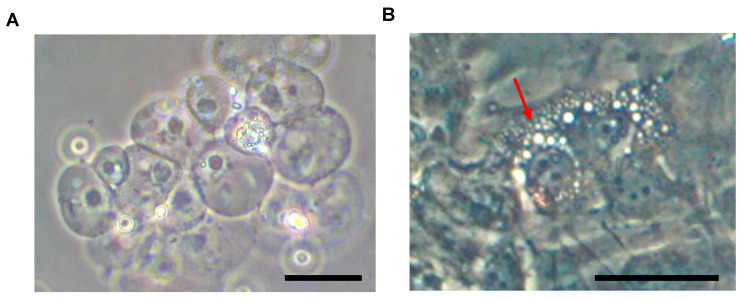
Murine NC cluster appearance following NP aspiration under microscopic observation (**A**). After plastic adherence (**B**), NCs display a characteristic vacuolated morphology (red arrow). Abbreviations: NC = notochordal cell; NP = nucleus pulposus. Scale bars = 50 μm.

In humans, from development until early postnatal life [6], the NP is characterized by large, vacuolated notochordal cells (NCs) (Figure 2). 

However, as early as soon after birth, NCs are gradually replaced by mature NPCs (formerly referred to as small chondrocyte-like cells [CLCs]). By late adolescence (around 17 years of age), only a few sparse NCs can be found, with mature NPCs accumulating due to the onset and advancement of intervertebral disc degeneration (IDD) [7,8]. It has been suggested that the morphological differences between NCs and mature NPCs represent different stages of cellular activity or differentiation, rather than two different lineages. In this context, all the cells residing within the NP may be considered to have differentiated along the notochordal lineage, with the morphological differences representing different physiological or pathological stages of cell aging and IDD [4].

The IVD’s avascular nature required notochordal-derived NPCs to develop distinctive characteristics in order to adapt to an acidic, hypoxic, and hyperosmotic environment [1]. In particular, NCs are involved in the constitution of an anti-angiogenic niche, through the secretion of an anti-angiogenic ECM and the release of anti-angiogenic factors [9]. The degree of GAG glycosylation has been shown to inhibit endothelial migration in vitro by providing the IVD with water-imbibing properties and high magnitudes of pressurization, which may hinder blood vessel ingrowth [10]. The ability of aggrecan to inhibit endothelial cell adhesion/migration and nerve outgrowth depends on the degree of glycosylation of GAGs, suggesting that chondroitin sulfate is largely responsible for proteoglycans’ anti-angiogenic effects [11]. Together with the chondroitin sulfate, the Noggin (Nog) factor inhibits endothelial cell invasion and tubular formation [12]. Fas ligand (FasL), expressed by normal NPCs, induces apoptosis in vascular endothelial cells, thereby inhibiting blood vessel infiltration [13]. Moreover, FasL also plays a key role in limiting the recruitment of immunogenic cells, including macrophages and lymphocytes which, combined with limited vascularization, make the healthy IVD one of the few largely immune-privileged tissues [14]. The thin CEPs anchor the IVD to the adjacent vertebral bones, serving as a selectively permeable barrier. This structure enables nutrient uptake into the IVD mainly through diffusion via the CEPs or from the restricted blood supply in the outer layers of the AF [9]. The avascular nature of the NP persists throughout life but may be compromised as part of the IDD cascade [9].

The embryonic development of the human NP provides invaluable clues to understanding its complex physiology and pathology. Unlike other species such as rodents, which retain NCs for up to half of their lifespan, human NCs start to be depleted early during fetal life and become undetectable by late childhood. The early loss of the NC population has been associated with the onset of IDD changes, thus suggesting the fundamental role of these cells in maintaining healthy NP homeostasis [15]. For this reason, it has also been speculated that NCs may serve as a promising cell source for IVD regeneration [16]. This review aims to investigate the complex process leading to the development of the NP from the embryonic notochord. The interplay among the plethora of transcription factors involved and the cellular changes occurring at different embryonic stages will be illustrated. Furthermore, the relevance of NP development to pathological changes, including both IDD and the aberrant development of malignant chordoma from notochordal remnants, will be discussed.

## 2. Embryonic Development of the NP

**Figure 3:**
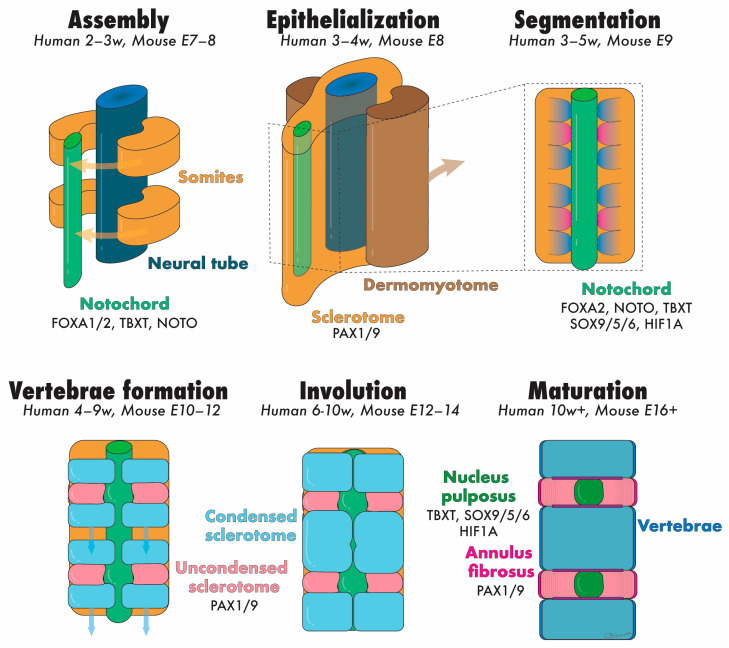
Schematic representation of the developmental stages of the NP and annotation of the transcription factors speculated to be involved. Abbreviation: E = embryonic day, NP = nucleus pulposus, and w = embryonic week. Colored text matches the tissues illustrated with identical colors. Created with Adobe Illustrator.

During embryogenesis, the IVD develops through the maturation of two key structures: the notochord and sclerotome (Figure 3). These components give rise to the NP and the surrounding tissues (i.e., AF, CEPs, vertebrae, and other vertebral soft tissues), respectively [17]. The notochord is a rod-like, transient structure that forms in the midline of chordates and vertebrates. In the third week of human gestation (equivalent to embryonic day [E]6.0 in mice), the initially single-layered blastocyst undergoes reorganization into a three-layered gastrula composed of endoderm, ectoderm, and mesoderm [18]. During this pivotal process, the cells of the dorsal organizer, situated at the midline of the gastrula, form a distinct structure from the adjacent mesoderm, known as the chordamesoderm (equivalent to E7.5 in mice) [19]. Through mediolateral intercalation and convergence towards the dorsal midline (i.e., convergent extension, occurring approximately between E8.5–10.5 in mice), chordamesoderm cells become vacuolated and arrange themselves in an elongated pile surrounded by a dense ECM rich in collagens and sulfated proteoglycans, forming the notochordal sheath. Importantly, the pressure within the amniotic cavity plays a critical role in orchestrating convergent extension and ensuring proper embryo development along the anterior–posterior axis [20]. 

Mechanical forces acting on the developing notochord at this stage are crucial for subsequent embryo elongation and locomotion development in various chordate organisms [18]. The osmotic pressure exerted within the vacuoles against the notochordal sheath confers upon the notochord its characteristic rod-like appearance [17]. The notochordal sheath is essential to contain the hydrostatic pressure within the notochord and guide NP development. Indeed, gene mutations affecting the development of the notochordal sheath, such as *SOX5*/*6* and *SHH*, may result in the complete failure of NP patterning in mice [21]. Similarly, smoothened (*SMO*) deletion from *SHH*-expressing murine cells prevents the notochordal sheath from being formed and the NP from developing within the IVD region [22]. However, *SMO* deletion after the formation of the notochordal sheath did not hinder NP development in a mouse model [23]. 

During gastrulation (equivalent to E8.0 in mice), the paraxial mesoderm, located at either side of the neural tube, gradually thickens to form the somitomeres, eventually undergoing segmentation and assembling into single units termed somites. This process is finely regulated temporally and spatially, proceeding in an anterior to posterior fashion along the embryo axis [17]. Soon after formation, in a process termed ephithelialization, each somite differentiates into two distinct tissues: the dermomyotome (which forms the skin of the back area and the underlying paraspinal muscles) and the sclerotome (which forms the AF, CEP, vertebrae, and other connective tissues of the spine) [24]. These events occur during the fourth week of human gestation [25]. A more detailed discussion of somite development is out of the scope of this review and has been extensively described elsewhere [17].

Around E13.5 in mice, the notochord initiates segmentation along the anterior–posterior axis, showing early signs of expansion into the IVD anlagen. The transition from the rod-like notochord and sclerotome to the respective NPs and vertebrae occurs simultaneously on E14.5 [26]. The notochord indeed plays a prominent role in this complex process. Recent evidence has suggested that notochord ablation impeded both vertebral and IVD segmentation in chick embryos. Conversely, sclerotome ablation prevented notochord segmentation and the further development of surrounding vertebral structures. These findings demonstrated that the notochord itself is not intrinsically segmented, and that the sclerotome orchestrates the correct development of NPs from the notochord, and the physiological assembly of the IVD and vertebral tissues from the sclerotome [27]. Intriguingly, regions where mesenchymal cells develop into vertebral bodies lack NCs, though seemingly not due to increased NC death [28]. 

Several models have been proposed to elucidate the transformation of the notochord into the NPs. According to the “pressure” model, NCs are “squeezed” away from the vertebrae towards the region where the NPs form. The osmotic swelling and resistance related to type II collagen fibrils within the middle region of the vertebral body may induce the migration of the notochord towards the IVD regions [29]. It has been previously shown that mutant mice lacking the notochordal sheath still presented normal vertebrae, although the notochord retained a rod-like structure and NCs were dispersed throughout the vertebrae [22]. Structural alterations of the notochordal sheath may also result in the failed segregation of the notochord from the vertebral bodies. Type II collagen is one of the major components of the notochordal sheath and is directly deposited by NCs into the surrounding ECM. In *COL2A1*-null mice, the notochord was not removed from the vertebral bodies and the IVDs did not develop, probably because of the inability of the notochordal sheath to withstand osmotic pressure. Furthermore, these animals showed enlarged vertebral bodies and abnormal collagen fibril deposition [29].

According to the “repulsion/attraction” model, the regions where NPs are supposed to develop may express attractant molecules that specifically recruit NCs to aggregate in NP-forming areas. Alternatively, repulsive signals may be expressed in vertebrae-forming regions, driving NCs away. Putative pathways involved in this mechanism include Eph/ephrin and Robo/Slit. The former has been described to set and maintain tissue borders and promote selective cell segregation in several areas of the developing embryo. Indeed, the EphA4 receptor has been localized in the notochord whereas ephrins have been identified in the surrounding mesenchyme. Therefore, these mediators may be essential to retain NCs where the NPs will eventually develop. Conversely, when Slit glycoproteins bind to Robo receptors, NCs tend to be repelled. Since Robo is expressed by mesenchymal cells and Slit by NCs, this may suggest an additional mechanism contributing to the segregation of the two cell types towards their respective tissues [26]. 

After week 10 (by E15.5 in mice), distinct NPs containing NCs and large vacuolated cells are identifiable [28]. Several different mechanisms have been identified to play a key role in the notochord-to-NP transition, including mechanical forces. In a recent study, the authors postulated that, at the early stage of NP development, the segmented notochord bulges exerting a bi-axial strain on the surrounding sclerotome, which may confer to the AF its typical cross-aligned collagen fiber orientation. Curiously, as notochord bulging does not occur in avians, these animals lack NPs and present a retained notochord surrounded by concentrically aligned collagen fibers [30].

## 3. Transcription Factors in NP Development

Transcription factors play a pivotal role in orchestrating the intricate developmental cascade during the embryogenesis of tissues and prospective organs [31]. These specialized proteins regulate gene expression by binding to specific DNA sequences, thereby modulating the rate of expression of target genes as a mean to guide cellular differentiation, proliferation, and tissue morphogenesis [31]. Through their precise control of gene transcription, transcription factors exert profound influence over the multifaceted pathways driving the embryogenic development of tissues, including the NP [32]. While the intricate regulatory mechanisms governing the NP remain elusive and warrant comprehensive further exploration, it is worthwhile to delve into the key transcription factors crucial for NP tissue development, as evidenced by genetic mouse studies.

*FOXA1* and *FOXA2*, which are expressed early in development, are intricately involved in regulating the spatiotemporal organization of the notochord. Specifically, they regulate the expression of other key transcription factors, i.e., *TBXT*, and Homeobox Gene Not (*NOTO*), and are thereby pivotal in controlling the morphogenesis of notochord formation and defining NC types, as evidenced by severe IVD deformities in respective knockout models [33]. Moreover, *FOXA1* and *FOXA2* are involved in the transition of NCs to NPCs. The aptly named factor *NOTO* is a highly conserved factor involved in left–right patterning and specifically expressed in the posterior notochord during gastrulation. It works downstream of other factors such as *TBXT* and *FOXA2*, though their specific functions remain obscure. Similarly, *TBXT*, under control of Shh, is crucial for notochordal development as well as other axial structures, and the deletion of *TBXT* during development prevents spinal formation [34,35,36]. Moreover, *TBXT* expression following development is involved in ECM production [37] and has been thereby identified as a pivotal marker for NPCs [4]. Both *NOTO* and *TBXT* have shown the capacity, when their expression is enhanced, to upregulate key NP ECM production genes [38,39].

*SOX5*, *SOX6*, and *SOX9* collectively contribute to NP and inner AF formation, with *SOX9* regarded as a master regulator in chondrogenesis, and directly promote type II collagen and aggrecan production. The absence of SOX9 results in complete cartilage absence, highlighting its indispensability in tissue development. *SOX5* and *SOX6* enhance *SOX9* function and are expressed in both the sclerotome and notochord during the critical developmental stages [40]. Alternatively, *PAX1* and *PAX9* can interfere with the activation of the aggrecan gene by SOX9 and SOX5/6 during development [41,42]. Through this mechanism, *PAX1*/*PAX9* with *SOX9*, *SOX5*, and *SOX6*, are pivotal in delineating the inner AF and outer AF regions of the disc [43,44]. This is achieved through a complex regulatory network that involves intricate positive and negative feedback loops, as well as their interconnected regulation with bone morphogenetic protein (BMP) and transforming growth factor (TGF)-β pathways. Finally, as the NP constitutes the largest avascular tissue in the human body [9], resident cells are subjected to oxygen and nutrient paucity, demanding metabolic alterations to support NPC survival [45,46]. A key transcription factor regulator to support this adaptation is HIF-1α, a regulator of glycolytic metabolism and suppressor of hypoxia-induced apoptosis [47]. The repression of *HIF1A* during development leads to severe cell death in the notochord and the complete disappearance of the NP, underlining its pivotal role in supporting cell survival in the hypoxic disc environment [48].

Understanding the regulatory mechanisms of these transcription factors provides invaluable insights into the complex developmental processes underpinning NP formation and maintenance. Notably, most observations are derived from murine embryos, as human samples are limited due to ethical concerns, and direct translation requires caution. Nevertheless, harnessing this knowledge may pave the way for novel therapeutic interventions targeting IVD-related pathologies and degenerative disorders. Moreover, due to their critical role in the development and maintenance of the NPC phenotype, these transcription factors are key markers to be used for assessments of healthy and diseased NPCs [4,49]. The main transcription factors involved in NP development are summarized in Table 1.

## 4. Cellular Composition of Notochord to NP

For humans, as development progresses, NCs gradually transition into mature NPCs. However, a clear consensus on the terminology for these different cell types is still lacking [15,60,61]. Mature NPCs share similarities with the chondrocytes of hyaline cartilage but exhibit distinct collagen-to-proteoglycan ratios, gene expression profiles, and biological responses [15,62,63,64], tailored to their unique roles in the IVD. Interestingly, inherent repair mechanisms involving local stem or progenitor cells have been documented, which participate in the maintenance and potential regeneration of the IVD. Understanding these transitions and the regulatory mechanisms of the cellular population transitions may not only shed light on developmental biology but also open avenues for therapeutic strategies in treating IVD-related diseases.

Lineage and phenotypical characterization studies have confirmed that mature NPCs and NCs are of the same descent [55,65], though others have hypothesized origins from inner AF or CEP tissues [66]. Nevertheless, due to their evident differences in cell function and morphology, it is valuable to differentiate between NPCs and NCs. Bach et al. recommended distinguishing embryonic NCs (eNCs), NCs, and NPCs; both eNCs and NCs present as vacuolated cells, but eNCs refer to the NCs populating the embryonic notochord, while NCs are the specific vacuolated cells of the NP within the developed IVD [67,68]. Moreover, eNCs have been suggested to present a single large fluid-filled vacuole, while more mature NCs exhibit fragmented vacuolation [60]. Both eNCs and NCs are highly positive for KRT-18/19, CD24, TBXT, CD55, and CDH2, among other key markers [4,60]. The NCs begin their transition towards mature NPCs prior to birth and fully transition by around ten years of age in humans [60,69]. This differs from species such as mice, rats, and non-chondrodystrophic dogs, which maintain their NCs into adulthood, and from horses, goats, or sheep, in which NCs have mostly receded at birth [60,69]. These developmental aspects are critical for selecting the appropriate animal models to assess IVD-related diseases or therapies, particularly considering the relatively high regenerative potential of NCs [60,70,71,72].

The transition from NCs to NPCs is poorly understood, yet it is speculated to potentially involve, in part, the transition of TIE2-expressing NP progenitor cells [73]. Induced pluripotent stem cell (iPSC) differentiation experimental work has highlighted the transition of NCs to mature NPCs involving a high rate of TIE2-expressing cell populations [74], and recent investigations have demonstrated that TIE2-enriched NPC populations exhibit cells with fragmented vacuolation [75]. These results suggest that TIE2-expressing NP progenitor cells function as a potential transitional cell population, although these observations have not been made in vivo. Moreover, TIE2+ NPCs have been observed in both human and canine NP fetal tissues [73]. Conversely, contradicting reports are available in mice, where cultured fetal mouse NPCs have shown high positivity for TIE2 [76]; however, developing mice transcriptomic studies failed to observe any TIE2+ cells within the NP [77]. Moreover, in humans, isolated TIE2+ NPCs are only detected up to early adolescence and show a sharp decline after 20 years of age. Additional stem-like or progenitor-like cells displaying properties similar to mesenchymal stromal cells have been identified in various species within the NP; however, these generally remain poorly defined [73]. Moreover, their role or place in the transition from NCs to NPCs remains unknown.

The maturation of NCs to NPCs is marked by the expression of GD2 and CD24 [4], and is characterized by a considerably smaller cell diameter. NPCs are usually present as single cells within the NP matrix [60], until IDD and aging promote these cells to form clusters. As part of the avascular and enriched water-binding proteoglycans in the NP, NPCs form a highly specialized population able to cope with harsh osmotic, hypoxic, low-glucose, and acidic environments. Key markers such as HIF-1α, tonicity-responsive enhancer binding protein (TonEBP), and GLUT-1 form key adaptations for this cell type to survive and allow for their active involvement in the continuous remodeling of the proteoglycan and collagen-rich NP [49,78,79]. Nevertheless, as part of aging, IDD, and wear and tear, a decline in the number of NPCs is observed, and a switch from active NPCs to senescent cells may occur [80], which may actively promote IDD and inflammation [81,82]. Moreover, the progression of IDD may in turn attract new immunogenic [14,83,84] and other homing cells [85,86] to integrate into the IVDs, thereby drastically changing the overall cellular composition of the NP.

Finally, though the general implication of NCs to mature NPCs suggests a straightforward transition, recent evidence indicates that this representation may be an oversimplification. Specific cell profiling assays, particularly single-cell RNA sequencing studies, have highlighted separate NPC populations present within the disc. Moreover, further distinctions between the types of NPCs and their differentiation pathways have been found to be linked to certain pathologies and symptoms [87,88,89]. For example, Jiang et al. [90] identified a specific MMP3+, SLC7A2+, and TM4SF1+ NPC clusters associated with symptomatic low back pain. In contrast, Han et al. [91] revealed up to seven diverse NPC populations. These included two specific populations, i.e., inflammatory NPCs, overexpressing chemokines such as CXCL2 and CXCL8, and hypertrophic NPCs strongly expressing MMPs, which were both found to be linked to IDD progression. Moreover, these IDD-associated populations appeared to play key roles in metal-ion transport and redox alterations, highlighting the role of oxidative stress in IDD. Complementary findings by Li et al. [92] identified five distinct NPC populations, with four of these populations showing an increased presence in IDD samples. They referred to these as calcification-inhibitory, calcification, inflammatory, and fibrogenic NPC populations, each characterized by unique markers. Overall, these studies illustrate that NPCs can likely be classified into distinct populations and have the ability to differentiate within the fully developed IVD. This process is further associated with the progression of IDD and low back pain. Notably, there are clear distinctions among the populations identified between different studies [87,88,89,90,91,92,93]. This warrants further careful investigations and calls for an evidence-based consensus within the spine community, providing a valuable basis for a future review.

## 5. The Impact of Aging and Degeneration on the NP

IDD is recognized as a major contributor to low back pain, a condition affecting millions and acknowledged as the primary cause of disability worldwide [94]. Yet, despite its staggering prevalence and socioeconomic impact, available therapies remain merely palliative. IDD involves complex biochemical and structural changes within the disc, leading to the reduced function of the IVD, thereby compromising the spine as a functional unit, which may promote pain and disability [1]. Observing the continuous development and changes, particularly the transformation of the NP cell population from NCs into mature NPCs and the impact of aging on this process, is crucial for comprehending the pathophysiology of IDD [95]. As previously detailed, the large vacuolated NCs are the primary cellular component of the embryonic NP, and represent a highly metabolically active cell type that produces copious amounts of proteoglycans [4]. The transition of NCs present at birth to post-natal mature NPCs coincides with a period of high cell death within the IVD [6]. Moreover, as part of the aging process, mature NPCs are worn down by several factors, e.g., high biomechanical forces [96], the accumulation of reactive oxygen species and oxidative stress [75], and (epi)genetic modifications [97], all of which can be further exacerbated by lifestyle factors and genetic predispositions [98,99,100], resulting in a gradual decline in the number and activity of NPCs [101,102]. This age-related decline is accompanied by significant alterations in the ECM, including a reduction in the proteoglycan content and an increase in collagen cross-linking. These changes lead to a decrease in the resilience and mechanical properties of the NP, making it more susceptible to IDD [103].

The proteoglycan depletion occurring with aging and IDD has been attributed to a combination of reduced synthesis by NPCs and increased degradation by proteolytic enzymes [104]. Specifically, aggrecanundergoes fragmentation and depletion, contributing to decreased hydration and altered biomechanical properties [1]. The collagen composition in the NP also changes. There is an increase in collagen cross-linking, particularly of type I collagen, which replaces type II collagen. This change in collagen composition contributes to the fibrosis and loss of elasticity observed in degenerative IVDs [29]. Matrix metalloproteinases (MMPs), particularly MMP-1, -3, -7, -13, and -14, are upregulated in IDD [105], and degrade various ECM components, including collagens and proteoglycans, thus compromising the NP structural integrity. A disintegrin and metalloproteinase with thrombospondin motifs (ADAMTS) enzymes, notably ADAMTS-4 and -5, are also implicated in aggrecan degradation. Their increased activity induces aggrecan fragmentation, contributing to proteoglycan loss and NP degeneration [106]. Pro-inflammatory cytokines such as interleukin (IL)-1β, IL-6, IL-17, tumor necrosis factor alpha (TNF-α), and interferon gamma (IFN-γ) are elevated in the degenerative NP. These cytokines stimulate MMP and ADAMTS expression, promoting ECM degradation and inflammation. Additionally, chemokines, including monocyte chemoattractant protein-1 (MCP-1), can attract immune cells to the otherwise immune-privileged NP, exacerbating inflammation and tissue damage [84].

**Figure 4:**
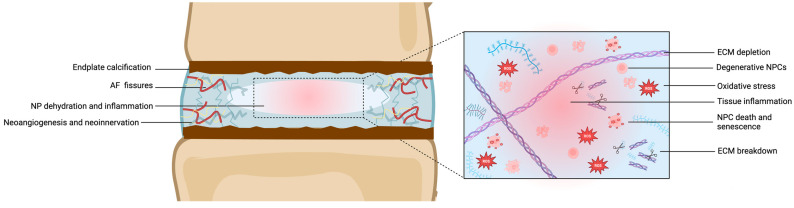
Schematic representation of IDD (**left**) and microscopic structure of the degenerative NP. Abbreviations: AF = annulus fibrosus; ECM = extracellular matrix; IDD = intervertebral disc degeneration; NP = nucleus pulposus; NPC = nucleus pulposus cells. ROS = reactive oxygen species. Created with BioRender.com.

Mature NPCs in the adult NP are less metabolically active than their embryonic counterparts and produce fewer proteoglycans. This results in a decrease in the hydration and gel-like properties of the NP, leading to a more fibrous and cartilaginous structure [63]. Further cellular changes can also occur as NPCs undergo senescence with aging and IDD, characterized by irreversible growth arrest and altered gene expression. Indeed, senescent NPCs secrete pro-inflammatory cytokines, MMPs, and reactive oxygen species, contributing to tissue inflammation and degradation [95]. Furthermore, the increased expression of apoptotic markers, such as cleaved caspase-3 and BAX, indicates apoptotic cell death in the aging NP, particularly in response to oxidative stress and pro-inflammatory cytokines [104]. Advanced glycation end products (AGEs) accumulate in the NP with age and IDD, leading to the increased cross-linking of matrix proteins. This cross-linking stiffens the ECM, impairing NP hydration and elasticity [107]. Finally, biomarkers of angiogenesis, including vascular endothelial growth factor (VEGF) and CD31, are also elevated in the aging NP. Increased neoangiogenesis correlates with IDD and vascular infiltration, exacerbating inflammation and matrix degradation. Neurotrophic factors like nerve growth factor (NGF) and substance P reflect neoinnervation of the aging NP. This nerve ingrowth is speculated to contribute to discogenic pain and inflammation [108]. These changes are depicted in Figure 4.

In short, the impact of aging on the NP structure and physiology is a complex process involving the developmental transitions and age-related changes in cellular activity and matrix composition. Understanding these processes is essential for the development of effective therapeutic strategies to prevent or treat age-related IDD and associated low back pain.

## 6. The Pathogenesis of Chordoma

Throughout IVD maturation, most of the notochord regresses, leaving behind the NP as its primary remnant in the adult spine. However, in some instances, NCs can persist in the distinct sites of the spine and proliferate abnormally, potentially leading to the development of chordoma. Chordoma is a rare and slow-growing bone tumor predominantly affecting the axial skeleton, with the sacrum and skull base being the most common sites. Specifically, approximately 50% of chordomas arise in the sacral region, 30% in the skull base, and 20% in other areas of the spine [109,110]. The preference for specific anatomical locations is believed to be linked to the persistence of notochordal remnants in these regions [111], predisposing individuals to chordoma development later in life [65]. Additionally, several studies have shown that TBXT is consistently expressed in chordomas, regardless of their anatomical location [36]. Therefore, increased TBXT expression serves as a hallmark of chordomas and is often used as a diagnostic marker for these specific tumors [112]. Although the pathogenesis of chordoma is not fully understood, TBXT expression in chordomas supports the theory that these tumors arise from remnant NCs that have undergone malignant transformation. Furthermore, the NC origin of chordoma cancer cells engenders specific cellular adaptations that complicate the treatment of chordoma. As NCs are resistant to hypoxia-related stress (in part due to high HIF-1α expression) and present metabolic adaptations to strive under harsh nutrient conditions, chordomas are inherently resistant. Markers such as HIF-1α and factors involved with metabolic regulation (e.g., GLUT-1) have been suggested as prognostic markers for overall chordoma survival [113,114]. Following its onset, chordoma typically exhibits slow growth, but may evolve into a locally aggressive tumor, infiltrating adjacent tissues and structures, including the spine, nearby nerves, and blood vessels [109]. Managing chordomas in regions such as the skull base and sacral area poses considerable challenges due to the complex anatomy and proximity to vital structures. Consequently, timely detection and intervention play a pivotal role in enhancing patient outcomes in chordoma cases. Effective treatment often necessitates a multidisciplinary approach, incorporating surgery, radiation therapy, and occasionally chemotherapy [109].

Achieving local control through surgical resection with adequate margins is a critical prognostic factor. However, as many tumors are located near vital structures, complete resection may be challenging and increases the difficulty of the procedure [111]. Despite the low radiosensitivity of chordomas, radiotherapy may be applied as an alternative or adjunctive treatment for tumors that are unresectable or challenging to resect completely [115]. Notably, carbon ion radiotherapy has been identified as an effective treatment option for unresectable chordomas in recent years [116]. Generally, chordomas are considered to be resistant to conventional chemotherapy, and no standard treatment regimen has been established. Currently, molecularly targeted therapies and immune checkpoint inhibitors are subjects of clinical research [111]. Previous studies report that the mean overall survival for patients with chordoma is 6.29 years, with survival rates of 67.6% at 5 years, 39.9% at 10 years, and 13.1% at 20 years. Aggressive modern surgical techniques have led to 10-year overall survival rates reaching 95% for skull base chordomas and ranging from 58% to 100% for chordomas of the mobile spine and sacrum [117]. Carbon ion radiotherapy achieves local control rates of 96%, 76%, and 54% at 1, 5, and 10 years, respectively. The overall survival rates for these intervals are 99%, 85%, and 69%, respectively [118]. Local recurrence and metastasis may occur during the prolonged course of the disease, necessitating careful and long-term follow-up [111].

## 7. Potential Therapeutic Strategies: Taking Inspiration from NP Development

As discussed, the development of the IVD involves a regulatory pathway with transcription factors, cell adjustments, and ECM changes (Figure 2). After birth, aging and stress lead to accumulating damage, compromising cellular integrity and ECM composition, which may lead IVD tissues to no longer be able to cope with biomechanical limits and potentially cause pain and disability [81,82]. Researchers are currently seeking novel therapies by leveraging the developmental processes of the IVD [60,119], as NCs are speculated to have notable regenerative potential [15,61,120]. Efforts target the integration of these cells and their associated matrisome into variable strategies for regenerating the IVD and tempering lower back and neck pain.

Cell therapy aims to either repopulate the NP with active ECM-producing cells or to rely on paracrine signaling from the injected cells to reactivate or home endemic cells to support IVD repair [16,85]. Among the various cell types under investigation for IVD regeneration [16], NCs have garnered scientific interest due to their unique regenerative properties [120]. These cells display high potency to instruct cells to take on active chondrogenic phenotypes resembling NPCs, temper inflammatory environments, and limit angiogenesis and nerve ingrowth [121,122,123]. However, challenges still exist as NCs are notoriously difficult to culture ex vivo with limited sources available and ethical considerations abound. However, advancements in reprogramming techniques present promising avenues, as elaborated upon below [124,125,126]. Alternatively, investigations are exploring NP progenitor cell transplantation for discogenic pain alleviation. These TIE2-expressing progenitor cells display stem cell-like traits, including enhanced proliferation, differentiation, paracrine activity, and ECM production [127,128,129]. Although these cells also face challenges regarding their maintenance and expansion, recent advancements in processing methods have supported their mass production with satisfactory phenotype retention [74,127,129,130,131]. The re-introduction of NP progenitor cells offers a compelling transplantation product, as they may theoretically constitute an unlimited source of regenerative cells within the NP environment [120].

Alternative strategies aim to employ cell-free methods to utilize the signaling factors produced by regenerative cell populations [132,133,134], particularly their extracellular vesicles (EVs) [135,136,137,138]. Research demonstrates that EVs derived from NCs contain potent bioactive molecules capable of modulating cellular behavior and are potentially able to promote tissue repair [139,140]. Furthermore, recent work has highlighted the therapeutic potential of EVs derived from TIE2-enhanced NPCs to reverse the induced IDD in a rat model [141]. These EVs outperformed those obtained from mesenchymal stromal cells as well as standard NPCs, indicating the potential benefits of drawing inspiration from embryonic sources.

Gene therapy and cell reprogramming techniques represent promising strategies for intervention in IDD [142,143]. Harnessing the regulatory power of notochordal and NP-related transcription factors, researchers have attempted to steer cellular differentiation towards phenotypes conducive to IVD regeneration. For example, the overexpression of *NOTO*, *TBXT*, or a combination of *NOTO* with *SOX9*, *SOX5*, and *SOX6* has been utilized to promote human iPSCs to take on an NC or NPC phenotype. These studies were able to show an increase in key NC/NPC markers and the production capacity of IVD-related ECM proteins [74,124,144]. In a recent in vivo study, iPSCs were induced to differentiate towards mesendoderm progenitor cells (MEPCs), precursors of NCs. Three different MEPCs doses were injected intradiscally 5 weeks after nucleotomy in a sheep model. One month after transplantation, MEPCs were shown to be viable, actively replicating, and expressing TBXT, KRT-8, -18, -19, and FOXA2 [145]. Alternatively, gene therapy strategies have used IVD development-related transcription factors to drive catabolic/senescent NPCs towards a more active and regenerative phenotype [146,147]. For example, the transfection of *TBXT* promoted an anabolic NPC phenotype in NPCs originally compromised by natural IDD [38]. These approaches highlight the anabolic potential of embryonically involved transcription factors to support the potential regenerative products against IDD (Table 1).

Finally, ongoing research explores the potential of notochordal tissue as a scaffold for IVD regeneration. This NC-derived matrix (NCM) is rich in bioactive factors and ECM, which shows promise as an instructive matrix to induce tissue repair and has demonstrated efficacy in treating IDD in preclinical models [148]. Studies indicate that NCM enhances ECM production, cell proliferation, and the expression of NPC markers [11,149]. While challenges in optimizing decellularization methods persist, decellularized NCM could potentially be used as a cell-free therapeutic agent or as a carrier [150,151] for cell-based therapies to support NP tissue repair [60].

In short, by capitalizing on the developmental mechanisms inherent to the NP, these diverse regenerative approaches hold significant promise for revolutionizing the treatment of IDD and associated pathologies. Through a multifaceted approach encompassing gene therapy, cell-based interventions, and biomimetic scaffolds, researchers aim to unlock the full regenerative potential, offering hope for improved outcomes and the quality of life for affected individuals. Nevertheless, these strategies are still in the early stages of development and will still have to overcome significant scientific, regulatory, and scalability hurdles before their widespread application in clinical settings [152,153,154].

## Figures and Tables

**Table 1:** Summary of the main transcription factors involved in orchestrating NP development.

Factors	Role in Embryogenesis	KO Mice [50]	Regulates	References
FOXA1/FOXA2	Spatiotemporal regulation NP development Activator of NC phenotype Regulates transition from NCs to NPCs	Single KO: no change Double KO: NP deformation/notochord fails to form Deformity most evident posteriorly High cell death in posterior somites Shorter tails	*NOTO* *TBXT*	[33,51,52]
TBXT	Regulates mesodermal precursor differentiation into NCs Supports maintenance of the notochord Regulates transition from NCs to NPCs Promotes aggrecan expression	Loss of somite formation	*NOTO*	[34,35,36]
NOTO	Regulates specification and formation of the notochord Left/right patterning	Moderate notochord malformations		[53,54,55]
SOX5/SOX6/SOX9	Regulates notochordal sheath formation Promotes transition from NCs to NPCs Defines the inner and outer AF region Promotes aggrecan and type II collagen expression	Severe chondrodysplasia Prevents proper segmentation Prevents NP formation Postnatal KO induces significant IDD	*PAX1/9*	[21,56,57]
PAX1/PAX9	Somite patterning Interferes with Sox5/6/9-induced aggrecan expression Defines the inner and outer AF region	No separation between vertebrae and discs Prevents sclerotome chondrogenesis Scoliotic-like deformity	*SOX5/6*	[41,42,44,58]
HIF-1α	Regulates the adaptation to hypoxia	Normal notochord development Reduced NP size (at E15.5) Fibrocartilaginous NP (at 1M) NPCs lacking vacuoles High cell death in NP		[48,59]

Abbreviations: AF = annulus fibrosus; IDD = intervertebral disc degeneration; KO = knockout; NC = notochordal cell; NP = nucleus pulposus; NPC = nucleus pulposus cell.

## Data Availability

Not applicable.
